# Computational Identification of Antigenicity-Associated Sites in the Hemagglutinin Protein of A/H1N1 Seasonal Influenza Virus

**DOI:** 10.1371/journal.pone.0126742

**Published:** 2015-05-15

**Authors:** Xiaowei Ren, Yuefeng Li, Xiaoning Liu, Xiping Shen, Wenlong Gao, Juansheng Li

**Affiliations:** 1 Department of Epidemiology and Health Statistics, School of Public Health, Lanzhou University, Lanzhou, Gansu, China; 2 Center for Statistics and Information, National Health and Family Planning Commission, Beijing, China; US Food and Drug Administration, UNITED STATES

## Abstract

The antigenic variability of influenza viruses has always made influenza vaccine development challenging. The punctuated nature of antigenic drift of influenza virus suggests that a relatively small number of genetic changes or combinations of genetic changes may drive changes in antigenic phenotype. The present study aimed to identify antigenicity-associated sites in the hemagglutinin protein of A/H1N1 seasonal influenza virus using computational approaches. Random Forest Regression (RFR) and Support Vector Regression based on Recursive Feature Elimination (SVR-RFE) were applied to H1N1 seasonal influenza viruses and used to analyze the associations between amino acid changes in the HA1 polypeptide and antigenic variation based on hemagglutination-inhibition (HI) assay data. Twenty-three and twenty antigenicity-associated sites were identified by RFR and SVR-RFE, respectively, by considering the joint effects of amino acid residues on antigenic drift. Our proposed approaches were further validated with the H3N2 dataset. The prediction models developed in this study can quantitatively predict antigenic differences with high prediction accuracy based only on HA1 sequences. Application of the study results can increase understanding of H1N1 seasonal influenza virus antigenic evolution and accelerate the selection of vaccine strains.

## Introduction

Influenza is an infectious disease caused by the influenza virus. Seasonal influenza causes approximately 250,000 to 500,000 deaths per year worldwide [1]. Influenza A/H1N1, A/H3N2, and B viruses are currently the main circulating subtypes of seasonal influenza that are included in each year’s influenza vaccine. After the 1918 Spanish influenza pandemic, the A/H1N1 influenza virus caused a number of outbreaks and epidemics until its disappearance from the human population in 1957 [2]. The H1N1 virus reappeared in the 1977 Russian influenza epidemic and began to exhibit seasonal circulation worldwide among the human population [3]. Vaccination is the most effective way to prevent influenza infection. The primary components of influenza vaccines have to be updated annually due to antigenic drift of influenza viruses. The influenza virus surface glycoprotein hemagglutinin (HA) is the primary target of neutralizing antibodies. The HA protein consists of two polypeptides, HA1 and HA2. The HA1 polypeptide plays a much more important role than HA2 in natural selection [4]. The gradual accumulation of amino acid substitutions in HA1 results in antigenic drift as antigenic properties change with time [5]. The seasonal influenza vaccine cannot offer effective protection against antigenically mismatched circulating strains. Antigenic differences between influenza strains are routinely measured by hemagglutination-inhibition (HI) assays that assess the ability of reference antiserum to prevent agglutination of red blood cells by influenza virus particles.

Smith *et al*. created an antigenic evolution map of influenza A/H3N2 virus using HI assay data and showed that antigenic evolution was more punctuated than genetic evolution, with a single amino acid change sometimes having a disproportionately large antigenic effect [6]. Li *et al*. also demonstrated that single HA mutations that alter both antigenicity and receptor binding avidity influenced influenza virus antigenic clustering [7]. Therefore, there are compelling reasons to identify antigenicity-associated sites in HA1. In turn, identification of these sites may offer insight into the antigenic drift of influenza viruses and accelerate the selection of vaccine strains. Sun *et al*. suggested that antigenicity-associated sites were not necessarily the same among subtypes of influenza A viruses because of different antibody binding sites [8]. For H3N2 seasonal influenza viruses, various studies have used statistical models to identify critical antigenic amino acid positions based on HI assays and HA1 sequences [9–12]. However, only a few studies have explored amino acid sites that drive the antigenic drift of H1N1 seasonal influenza virus using HI assay data. Recently, Huang *et al*. identified 41 H1N1 HA natural epitope residues based on 1572 HA sequences and 197 pairs of HA sequences with HI assays. Likelihood ratios used to correlate a single mutation at the amino acid position with an antigenic variant were utilized to establish the antigenic variant score [13]. However, recent studies have revealed that amino acid interactions play a key role in the process that antigenic site mutations modulate receptor-binding site (RBS) properties; hence, it is necessary to consider the joint effects of amino acid residues on antigenic drift when inferring the relationships between antigenic variants and amino acid changes [14].

In the present study, two widely used multivariate feature selection methods, Random Forest Regression (RFR) and Support Vector Regression based on Recursive Feature Elimination (SVR-RFE), were used to analyze the associations between amino acid changes in the HA1 polypeptide and antigenic variation based on HI assay data and HA1 sequences. These models identified the best combinations of amino acid sites that could quantitatively predict antigenic differences between different strains of H1N1 seasonal influenza with high prediction accuracy using HA1 sequences. Antigenic cartography methods were used to quantify and visualize the antigenic evolution of H1N1 seasonal influenza viruses from 1977 to 2008, and antigenic cluster transitions were found to be associated with important amino acid substitutions in the HA1 domain of HA. Finally, our computational approaches were validated with the H3N2 dataset to assess the performance when applied to H3N2 influenza viruses. We believe that the identification of antigenicity-associated sites will be helpful in better understanding the antigenic evolution of H1N1 seasonal influenza virus and facilitating influenza vaccine strain selection.

## Materials and Methods

### HI assay data

The HI assay data on the H1N1 seasonal influenza viruses used in this study were collected from related articles and documents published by the US Centers for Disease Control and Prevention and other collaborating centers of the World Health Organization (WHO) [15–22]. Under the premise of without changing the ratios between homologous and heterologous antibody titers, which were used to calculate antigenic distances, homologous antibody titers were standardized to equal 1280. The combined HI table was shown in S1 Table. The HI assay dataset contained 154 pairwise comparisons of 37 H1N1 viruses isolated from 1977 to 2008. The antigenic difference between two viruses was measured using the Archetti-Horsfall antigenic distance metric, which could control for receptor binding avidity variation between viral strains [7, 23]. The antigenic distance metric between strains A and B was defined using the following formula:

$$d(A,B)={\text{log}}_{2}\left(\sqrt{\frac{H^{AA}H^{BB}}{H^{BA}H^{AB}}}\right)$$ (1)

*H* ^*XY*^ = HI titer of virus strain *X* (antigen) relative to antiserum raised against virus strain *Y*. *H* ^*AA*^ and *H* ^*BB*^ = homologous titers of two strains. *H* ^*BA*^ and *H* ^*AB*^ = heterologous titers against each other.

Antigenic distances greater than 2 were usually treated as antigenic variants [9].

### HA sequence data

Thirty-seven HA1 amino acid sequences of H1N1 seasonal influenza viruses isolated from 1977 to 2008 were downloaded from the National Center for Biotechnology Information Influenza Virus Resource (Table 1) (http://www.ncbi.nlm.nih.gov/genomes/FLU/FLU.html) [24]. All HA1 sequences were aligned with ClustalW and trimmed to the same length (327 amino acid residues) [25]. Each pairwise amino acid sequence alignment was converted to a 1 x 327 vector. For a specific HA1 sequence position, the value was denoted as 1 if the residue types of the two HA1 sequences were different (mutation); otherwise, its value was denoted as 0 (no mutation). One hundred and fifty-four pairwise amino acid sequence alignments were linked with pairwise antigenic distances for further analysis.

**Table 1:** Full names and accession numbers of H1N1 seasonal influenza viruses from 1977 to 2008.

Full name	Accession number
A/WUHAN/371/95	CAC86625
A/BAYERN/7/95	CAD29944
A/BEIJING/262/95	ACF41867
A/BRAZIL/11/78	ABO38065
A/BRISBANE/59/07	ACA28844
A/BRISBANE/193/2004	ACD37424
A/CAMBODIA/0371/2007	ACI45444
A/CHILE/1/83	ABO38340
A/FLORIDA/13/07	ACF40117
A/FUKUSHIMA/141/2006	ACM17297
A/HONG_KONG/2652/2006	ACD37439
A/INDIA/6263/80	ABO38362
A/JIANGXI/160/2005	ACF76722
A/JOHANNESBURG/82/96	CAD29943
A/KENTUCKY/1/2005	ABI96135
A/KENTUCKY/02/2006	ABU86800
A/NEW_CALEDONIA/9/2004	ABQ09837
A/NEW_CALEDONIA/20/99	AFO65027
A/PHILIPPINES/673/2006	ACD37433
A/SHENZHEN/227/95	AAP34325
A/SICHUAN/4/88	AAA43231
A/SINGAPORE/6/86	ABO38395
A/SINGAPORE/14/2004	ABQ09838
A/SOLOMON_ISLANDS/03/2006	ABU50586
A/SOUTH_DAKOTA/06/2007	AFM72510
A/TAIWAN/1/86	CAA35097
A/TEXAS/36/91	ABD60955
A/USSR/90/77	AFM73477
A/VICTORIA/500/2006	ABQ09960
A/VIRGINIA/01/2006	ABI96152
A/ENGLAND/333/80	X00031
A/CHILE/4795/00	AFO66147
A/FUJIAN/156/00	AFQ90525
A/HONG KONG/1870/2008	AFM72587
A/MALAYSIA/100/2006	ABQ09959
A/MOSCOW/13/98	AFQ90529
A/NEIMENGGU/52/2002	AFQ90530

### Validation data

The previously published A/H3N2 dataset by Smith *et al*. was used to validate the biological relevance of the results of our approaches [6]. The validation dataset consisted of 271 pairwise antigenic distances among 52 viruses isolated between 1968 and 2003. The HI table was shown in S2 Table.

### Random forest regression

Random forests is a widely used machine learning algorithm that has been applied to classification and regression problems. It was introduced by Breiman in 2001 [26]. RFR is an ensemble of regression trees in which each tree is constructed on a bootstrap sample that is a subset of the original sample. At each splitting node of a tree, the candidate set of variables is a random subset of the explanatory variables. To reduce bias, no pruning step is performed; hence, all trees of the forest are maximal trees. The overall prediction of the forest is the average of predictions from all individual trees. For each tree, approximately one third of the data that are not included in the bootstrap sample are termed the “out-of-bag” (OOB) sample. The OOB sample is used as a testing set for that tree to estimate the prediction performance and then to evaluate variable importance. There are three parameters that need to be determined for RFR: *ntree*—the number of trees in the forest; *nodesize*—the minimum size of the terminal nodes; and *mtry*—the number of variables randomly sampled as candidates at each split. In this study, the parameters *ntree* and *nodesize* were set to their default values (*ntree* = 500 and *nodesize* = 5 for regression). The most important parameter (*mtry*) was tuned to achieve optimal predictive performance and increase the statistical power of the algorithm to detect true antigenicity-associated sites. Cases of *mtry* equaling *p* (equivalent to bagging), 2/3*p*, *p*/2, *p*/3, *p*/4, and sqrt(*p*) were considered, where *p* was the number of variables in the data set. The permutation-based “mean of squared residuals (MSE) reduction” was used as the random forest importance criterion to give a ranking of variable importance, and the variables with high ranks were considered as potentially associated with antigenicity. In this study, a variable reduction wrapper algorithm was employed to find the best putative combinations of amino acid sites [27]. This measure of importance was used to rank the variables from most to least important, and the RFR model’s performance could be monitored as more and more of the least important variables were iteratively removed. A 5-fold cross-validation (CV) procedure was used to evaluate the predictive performance of the RFR model. In the variable selection process, variable importance was not recalculated at each step because Svetnik *et al*. *2004* reported that severe overfitting results from recalculating variable importance. The CV test set MSE was used to assess the predictive performance of the RFR model. The smaller the MSE, the better the performance will be. The final RFR model was chosen by using the corrected Akaike information criterion (AICc) [28] to prevent overfitting. The RFR algorithm and the nested cross-validation procedure were implemented by the randomForest R package [29].

### Support vector regression based on recursive feature elimination

A support vector machine is a supervised data mining method based on statistical learning theory used for classification and regression. The basic idea of support vector regression was formulated by Vapnik *et al*. in 1995 [30]. Instead of minimizing the observed training error in the traditional regression procedure, support vector regression attempts to minimize the generalized error bound to achieve performance in which the generalization error bound is the combination of the training error and a regularization term that controls the complexity of the hypothesis space [31]. SVM-RFE is a popular embedded feature selection method. It was first proposed by Guyon *et al*. in 2002 to perform gene selection for binary classification problems [32]. SVM-RFE uses weight magnitude as the ranking criterion and generates a ranking of features using the backward feature elimination procedure, which starts with all the features and then recursively removes one or more of the least important features at a time. In this study, the ε-SVR-RFE algorithm was used to select a subset of amino acid sites that could contribute the most to the antigenic variation of H1N1 seasonal influenza virus as an alternative machine learning method to the RFR model. The 5-fold cross-validated MSE was adopted to evaluate the prediction performance of SVR models as more and more of the least important amino acid sites were recursively removed. The SVR model with the smallest AICc value was considered as the final selected model. The parameter ε and kernel function in SVR-RFE were set to 0.01 and radial basis function (RBF) kernel, respectively. The cost parameter *C* and parameter γ of the RBF kernel were optimized by extensive grid search. The search was conducted in the following ranges: *C* − 2^–8^ to 2^8^ with step size 0.1; γ − 2^–5^ to 2^5^ with step size 0.1. A 5-fold cross-validation was used in our experiments to determine optimum parameters. The SVR algorithm was implemented by the Matlab version of LibSVM provided by Chang and Lin [33]. The parameter optimization function was implemented using the Matlab toolbox Libsvm-FarutoUltimate3.1 provided by Li [34]. SVR-RFE was written in Matlab.

### Antigenic cartography

The quantitative analyses of the antigenic properties of H1N1 seasonal influenza viruses collected from 1977 to 2008 were performed using antigenic cartography methods as described previously by Smith *et al*. for human H3N2 viruses [6]. The HI assay data were used to construct two-dimensional (2D) antigenic maps in which the distance between points represents the antigenic distance as measured by a HI assay. One unit of antigenic distance on the antigenic map corresponds to a two-fold difference in the serological assay. The web-based software for Antigenic Cartography is available at http://www.antigenic-cartography.org/.

## Results

### Data set

Amino acid changes were identified in 77 positions among the 154 pairwise comparisons of H1N1 HA1 sequences by excluding 250 positions that had no mutations (S3 Table). The amino acid positions that underwent the same change (correlation coefficient = 1) were noted as one variable. These 77 positions were classified into 60 variables based on their collinearity. Among the 154 pairwise comparisons, 83 (54%) had an antigenic distance ≥ 2 (i.e., antigenic variant), and 71 (46%) had an antigenic distance < 2 (i.e., similar antigenicity). The H3N2 validation dataset was treated in the same way. Sixty-eight variables that were within positions 109 to 301 of the HA1 region according to Koel *et al*. were obtained [35].

### Antigenicity-associated sites identified using the RFR algorithm

**Fig 1:**
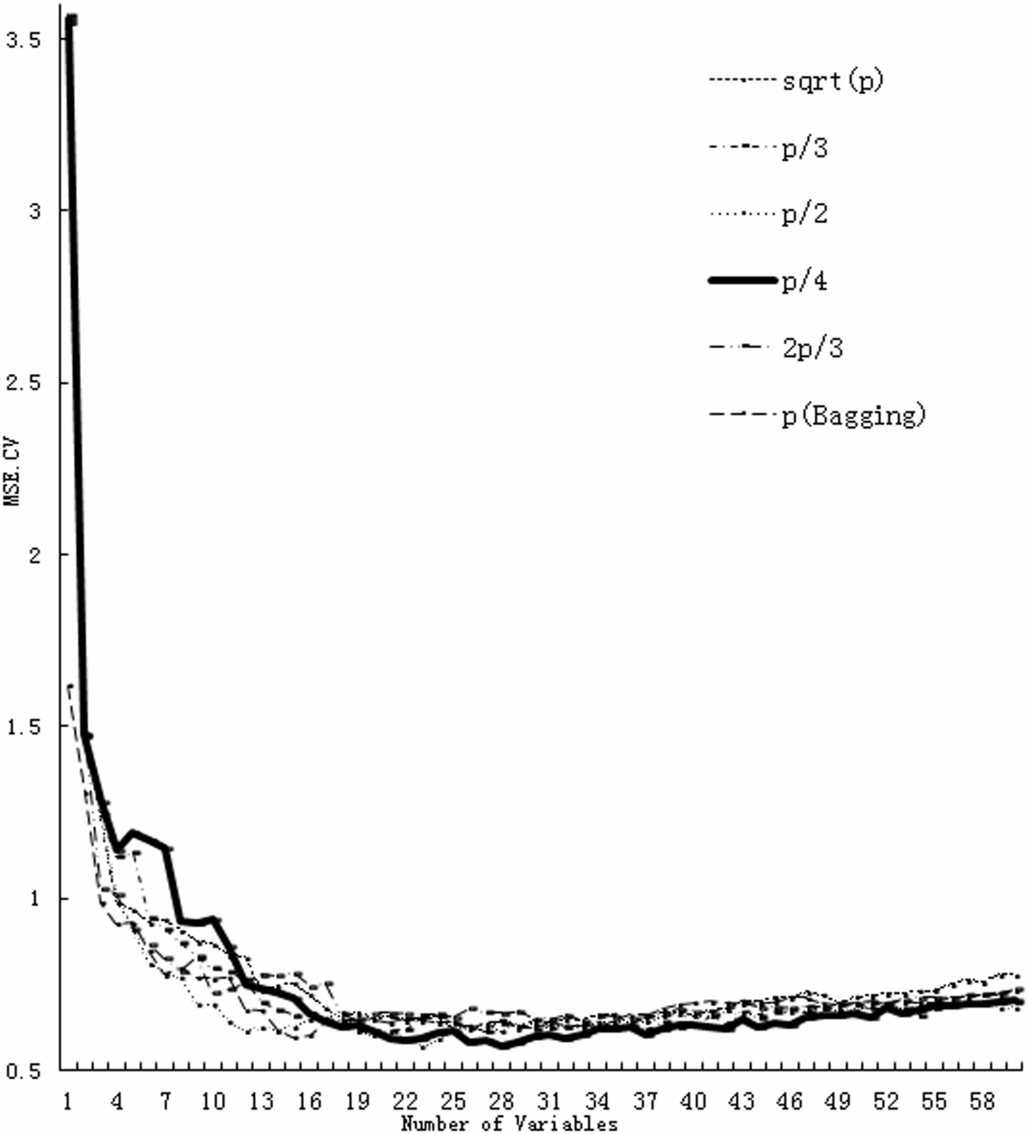
The cross-validation MSE of RFR against the number of variables selected using different *mtry* functions.

RFR was used to quantitatively correlate amino acid changes in the HA1 region with antigenic distances and to identify the best combinations of amino acid sites that contributed to antigenic drift of H1N1 seasonal influenza viruses. The CV test set MSE curve based on the variation of the number of variables selected was plotted for different choices of *mtry* (Fig 1). The plot showed that *mtry*, *p/4*, performed the best, but the other choices were nearly as robust. The results suggested that the RFR model with 18 variables (*mtry* = *p/4*) achieved the smallest AICc value of 0.8115. The predictive proportion of variance explained by the final RFR model was 0.8353, suggesting that the model achieved a good predictive performance. In Table 2, the 18 variables ranked in order of importance for the final RFR model were reported. Twenty-three amino acid positions were identified as responsible for the antigenic variation of H1N1 seasonal influenza viruses.

**Table 2:** Antigenicity-associated sites of H1N1 identified using the random forest regression algorithm.

Order of variable importance	Amino acid position		
1	141		
2	130		
3	43		
4	54	127	193
5	186		
6	80	271	
7	71		
8	36		
9	190		
10	194		
11	163		
12	128		
13	187		
14	189		
15	125		
16	121	205	
17	321		
18	133	191	

### Antigenicity-associated sites identified using the SVR-RFE

**Fig 2:**
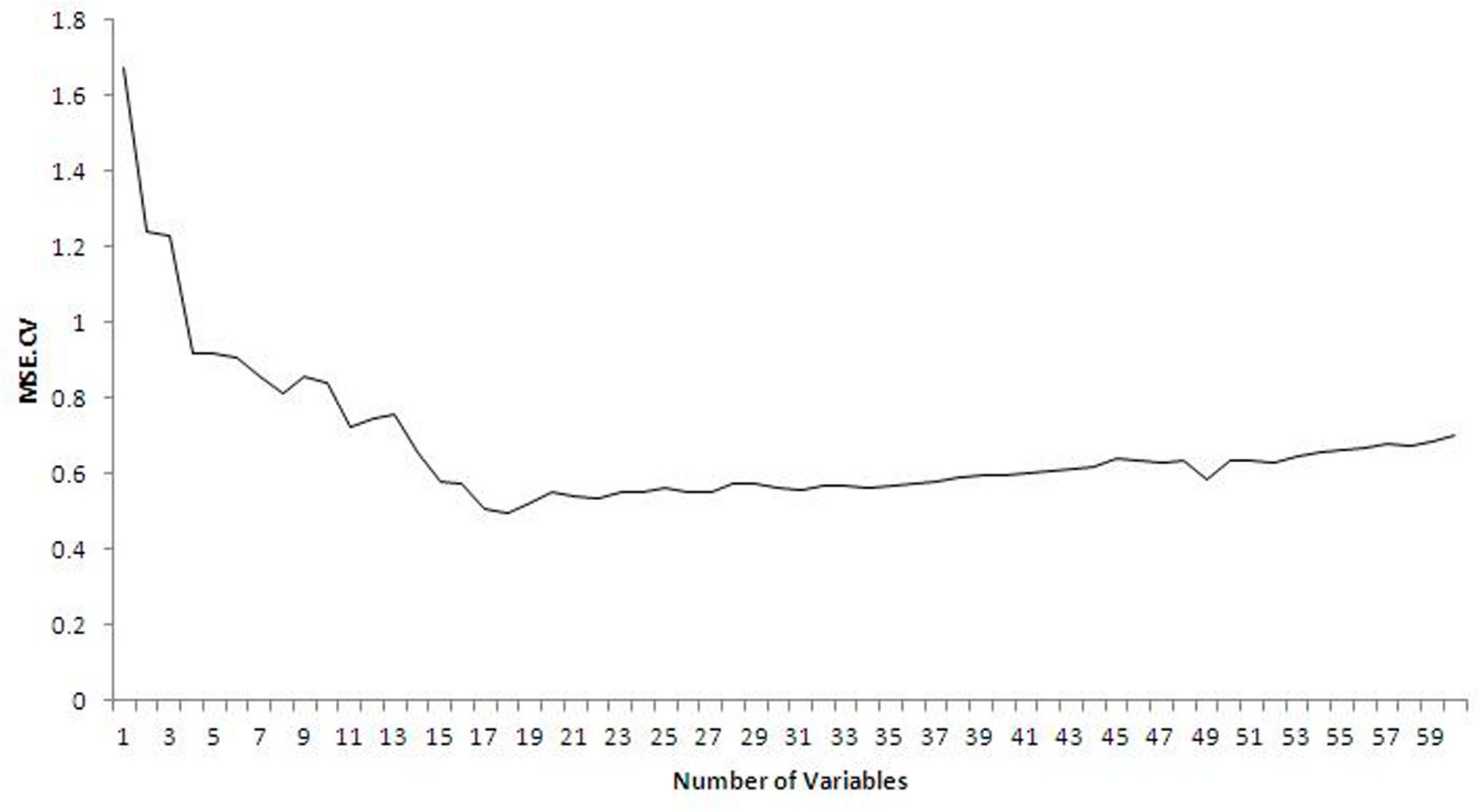
The cross-validation MSE of SVR against the number of variables used.

The SVR-RFE algorithm was performed to find the subset of amino acid residues that contributed the most to antigenic variation of H1N1 seasonal influenza virus. Fig 2 shows the cross-validation MSE against the number of variables used at each step of removing the least important variable. The minimum AICc value of 0.5826 was obtained at the 17-variable level by the SVR model (RBF kernel with parameters *C* and γ optimized to 4.5948 and 0.1015, respectively). The predictive proportion of variance explained rose up to 0.8610, which was 0.0257 higher than the RFR model. The final variable subset selected by the SVR-RFE algorithm is listed in Table 3 in order of variable importance. Twenty sites were identified as the best combinations of amino acid sites that could drive the antigenic drift of H1N1 seasonal influenza viruses.

**Table 3:** Antigenicity-associated sites of H1N1 identified using the support vector regression based on recursive feature elimination.

Order of variable importance	Amino acid position		
1	130		
2	54	127	193
3	43		
4	141		
5	190		
6	160		
7	121	205	
8	71		
9	273		
10	321		
11	125		
12	96		
13	277		
14	69		
15	187		
16	269		
17	57		

### Antigenic map of human H1N1 seasonal influenza viruses from 1977 to 2008

**Fig 3:**
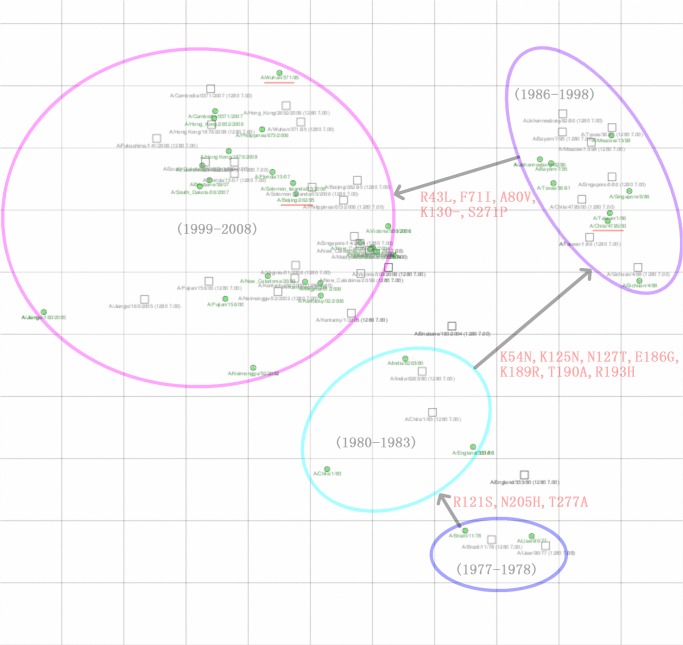
Antigenic map of human H1N1 seasonal influenza viruses from 1977 to 2008. The relative positions of strains (green circles) and antisera (uncolored squares) are adjusted such that the distances between strains and antisera on the map represent the corresponding HI measurements with the least error. One unit (grid) corresponds to a two-fold dilution of antiserum in the HI assay. The cluster-transition amino acid substitutions are shown in red.

Antigenic cartography methods were used to map the antigenic evolution of human H1N1 seasonal influenza viruses from 1977 to 2008 (Fig 3). The antigenic evolution of human H1N1 seasonal influenza viruses was significantly slower than the antigenic evolution of human H3N2 viruses when compared with the map published by Smith *et al*. The antigenic evolution of H1N1 seasonal influenza viruses appeared to be punctuated rather than gradual. Two large jumps were observed in the antigenic phenotype from the ‘80–83’ strains to the ‘86–98’ strains and from the ‘86–98’ strains to the ‘99–08’ strains, and one small jump was observed from the ‘77–78’ strains to the ‘80–83’ strains. Fig 3 summarizes the cluster-transition amino acid substitutions that are most likely responsible for the antigenic differences between antigenic clusters.

### Model Validation with the H3N2 dataset

Our proposed RFR and SVR-RFE approaches identified 12 and 18 antigenicity-associated sites of H3N2 influenza viruses, respectively. The importance of each variable was assessed. The variables ranked in order of importance for the RFR model and the SVR-RFE model were reported in Tables 4 and 5, respectively. The amino acid substitutions at seven positions (positions 145, 155, 156, 158, 159, 189, and 193), which were responsible for A/H3N2 antigenic cluster transitions over the 35-year period, had been experimentally validated by Koel et al.[35]. The top ten ranked variables recognized by the RFR model included the seven experimentally validated sites, while there were six experimentally validated sites within the top ten ranked variables identified by the SVR-RFE approach. Only one experimentally validated site, position 156, was not recognized by the SVR-RFE algorithm. Both of positions 133 and 135 that had been experimentally validated as the accessory substitutions by Koel *et al* were also respectively identified by both our approaches. Then the experimentally validated seven positions were used to construct the RFR and SVR model. The predictive proportions of variation explained by the 7-features RFR and 7-features SVR model were, respectively, 0.8447 and 0.8496. The high rankings of experimentally validated positions in both subsets and their significant contribution to explaining variation confirmed the effectiveness of our proposed approaches in capturing important antigenicity-associated sites and predicting antigenic changes.

**Table 4:** Antigenicity-associated sites of H3N2 identified using the random forest regression algorithm.

Order of variable importance	Amino acid position
1	**189**
2	*133*
3	**193**
4	**145**
5	**155**
6	144
7	**159**
8	216
9	**156**
10	**158**
11	163
12	*135*

Experimentally validated sites were marked as bold. Accessory substitutions were marked as italic.

**Table 5:** Antigenicity-associated sites of H3N2 identified using the support vector regression based on recursive feature elimination.

Order of variable importance	Amino acid position	
1	**189**	
2	*133*	
3	**155**	
4	**193**	
5	**158**	
6	216	
7	144	
8	285	
9	**159**	
10	**145**	
11	137	
12	196	
13	129	132
14	271	
15	131	
16	175	
17	*135*	

Experimentally validated sites were marked as bold. Accessory substitutions were marked as italic.

## Discussion

The WHO annually recommends an influenza vaccine composition for the coming influenza season. Antigenic characterization based on the HI assay, which is labor-intensive and time-consuming, is the primary procedure for influenza vaccine strain selection. It is of considerable importance to detect new antigenic changes occurring in HA protein when updating vaccine compositions. The punctuated nature of antigenic evolution of influenza virus suggests that a relatively small number of genetic changes or combinations of genetic changes may drive changes in antigenic phenotype [35].

In this study, RFR and SVR-RFE were used to identify amino acid sites associated with HA antigenicity of H1N1 seasonal influenza viruses on the basis of HI assay data and HA1 sequences. The methods used in the study are different from those of previous studies because the algorithms consider the joint effects of amino acid residues on antigenic drift when deriving antigenicity-associated sites. The results showed that CV test set MSE curves decreased sharply at first and then increased slowly with respect to the number of variables used. This trend might be attributed to the combined effects of amino acid substitutions [36].

The RFR and SVR-RFE algorithms identified 23 and 20 antigenicity-associated sites, respectively. Interestingly, thirteen amino acid residues overlapped between these two amino acid subsets. The first four ranked variables recognized by these two approaches contained the same six amino acid positions (43, 54, 127, 130, 141 and 193). With the RFR and SVR-RFE approaches, 2 and 5 new antigenicity-associated residues were identified, respectively, that were not covered by the natural epitope residues proposed by Huang *et al*. (Table 6). From the antigenic map of human H1N1 seasonal influenza viruses from 1977 to 2008, 15 amino acid positions were found that were likely to contribute to the antigenic difference between clusters. Surprisingly, the 23 and 20 antigenicity-associated sites cover 93.33% (14/15) and 73% (11/15) of cluster-differentiating mutations, respectively. The high ratio overlap reveals that the models used in this study are reliable. However, only 7 of these 15 cluster-difference substitutions overlapped with the five antigenic epitope regions (Sa, Sb, Ca1, Ca2, and Cb) previously described by Brownlee and Fodor [37]. The low consistency reflects the different recognition mechanisms of antigenic epitopes between natural selection at the population level and neutralizing monoclonal antibodies (MAbs) in the laboratory. There were also limited antigenic differences between the ‘99–06’ strains and the ‘06–08’ strains, which might be due to a single amino acid substitution at position 141 [35, 38, 39, 40].

**Table 6:** Comparison of amino acid positions related to antigenic variation of H1N1 seasonal influenza viruses identified by current and previous studies.

RFR^a^	SVR-RFE^b^	natural epitope residues^c^	antigenic cartography^d^	antigenic epitope regions^e^
		35		
36		36		
43	43	43	43	
		47		
54	54	54	54	
	57			
	69	69		
71	71	71	71	Cb
		73		Cb
80		80	80	
		82		
		94		
	96			
121	121	121	121	
125	125	125	125	Sa
127	127	127	127	
128		128		
130	130	130	130	
133		133		
141	141	141		Ca2
		146		
		153		Sa
	160	160		Sa
163		163		Sa
		183		
186		186	186	Sb
187	187			Sb
189		189	189	Sb
190	190	190	190	Sb
191		191		Sb
193	193	193	193	Sb
194		194		Sb
205	205	205	205	Ca1
		209		
		216		
		222		Ca2
		224		
		267		
	269			
271		271	271	
	273	273		
		274		
	277	277	277	
		295		
		310		
321	321			

^a^Twenty-three antigenicity-associated sites identified by random forest regression.

^b^Twenty antigenicity-associated sites identified by support vector regression based on recursive feature elimination.

^c^Forty-one natural epitope residues identified by Huang *et al*. [13].

^d^Fifteen cluster-difference substitutions revealed by antigenic cartography.

^e^Five antigenic epitope regions described by Brownlee and Fodor [37].

The H3N2 dataset was used to further validate the biological relevance of the results of our approaches. The RFR approach has been shown to be more effective in identifying the experimentally validated sites than the SVR-RFE algorithm, but the SVR model demonstrated better predictive performance compared to the RFR model. The final models developed in this study can perform quantitative prediction of antigenic differences between different strains of H1N1 based only on HA1 sequences and serve as an initial screening tool for antigenic variants before the labor-intensive and time-consuming HAI assay.

In conclusion, the proposed RFR and SVR-RFE approaches, which consider the joint effects of amino acid residues on antigenic drift, identified 23 and 20 antigenicity-associated sites of HA1 of H1N1 seasonal influenza viruses, respectively. The results obtained in this study can aid in better understanding of the antigenic evolution of H1N1 seasonal influenza viruses and accelerate the selection of vaccine strains.

## Supporting Information

S1 Table The combined H1N1 HI table. (XLS)

S2 Table The H3N2 HI table. (XLS)

S3 Table Amino acid changes in HA1 of H1N1 seasonal influenza viruses from 1977 to 2008. (DOC)
