# Supplementary material for: Computational Identification of Antigenicity-Associated Sites in the Hemagglutinin Protein of A/H1N1 Seasonal Influenza Virus
Source: PLoS One. 2015 May 15;10(5):e0126742. doi: 10.1371/journal.pone.0126742 (PMC4433265; doi:10.1371/journal.pone.0126742)
Supplement: S3 Table — (DOC) [file pone.0126742.s003.doc]

**S3 Table.** **Amino acid changes in HA1 of H1N1 seasonal influenza virus from 1977 to 2008.**

| **Virus strain** | **Amino acid position in HA1***^a^* | | | | | | | | | | | | | | | | | | | | | | | | | | | | |
| --- | --- | --- | --- | --- | --- | --- | --- | --- | --- | --- | --- | --- | --- | --- | --- | --- | --- | --- | --- | --- | --- | --- | --- | --- | --- | --- | --- | --- | --- |
|  | 35 | 36 | 40 | 43 | 47 | 54 | 56 | 57 | 66 | 68 | 69 | 71 | 73 | 74 | 80 | 82 | 83 | 85 | 86 | 94 | 96 | 111 | 120 | 121 | 125 | 127 | 128 | 130 | 133 |
| A/WUHAN/371/95 | D | S | K | L | I | N | S | V | E | E | S | I | K | E | V | T | P | P | E | Y | A | F | E | S | N | T | V | -*^b^* | T |
| A/BAYERN/7/95 | .*^c^* | . | . | R | T | . | . | . | . | . | . | F | . | . | A | . | . | . | . | . | . | . | . | . | . | . | . | K | . |
| A/BEIJING/262/95 | . | . | . | . | . | . | . | . | . | . | . | . | . | . | . | . | . | . | . | . | . | . | . | . | K | . | . | - | . |
| A/BRAZIL/11/78 | . | . | . | R | . | K | . | I | . | . | . | F | . | K | A | . | . | S | . | . | . | . | . | R | K | N | I | R | . |
| A/BRISBANE/59/07 | N | . | . | . | . | . | . | . | . | . | L | . | . | . | . | K | . | . | . | H | . | . | . | . | . | . | . | - | S |
| A/BRISBANE/193/2004 | . | . | . | . | . | . | . | . | . | . | V | . | . | . | . | . | . | . | . | . | . | . | . | . | . | . | . | - | S |
| A/CAMBODIA/0371/2007 | . | N | . | . | . | . | . | . | . | . | L | . | . | . | . | R | . | . | . | H | . | . | . | . | . | . | . | - | S |
| A/CHILE/1/83 | . | N | . | K | . | K | . | I | . | . | . | F | . | K | A | . | . | S | . | . | . | . | . | . | K | N | . | K | . |
| A/FLORIDA/13/07 | N | . | . | . | . | . | . | . | . | . | L | . | . | . | . | K | . | . | . | H | . | . | . | . | . | . | . | - | S |
| A/FUKUSHIMA/141/2006 | . | . | . | . | . | . | . | . | . | . | L | . | R | . | . | K | . | . | . | H | . | . | . | . | . | . | T | - | S |
| A/HONG_KONG/2652/2006 | . | N | . | . | . | . | . | . | . | . | L | . | . | . | . | K | . | . | . | H | . | . | . | . | . | . | . | - | S |
| A/INDIA/6263/80 | . | . | . | R | . | K | . | I | . | . | . | V | . | K | A | . | . | S | . | . | . | . | . | . | K | N | . | R | . |
| A/JIANGXI/160/2005 | . | . | . | . | . | . | . | . | . | . | L | . | . | . | . | . | . | . | . | . | . | . | . | . | . | . | . | - | S |
| A/JOHANNESBURG/82/96 | N | . | . | R | T | . | . | . | . | . | . | F | . | . | A | . | . | . | . | H | . | . | . | . | . | . | . | K | . |
| A/KENTUCKY/1/2005 | . | . | . | . | . | . | . | . | . | . | L | . | . | . | . | . | . | . | . | . | . | . | . | . | . | . | . | - | S |
| A/KENTUCKY/02/2006 | . | . | . | . | . | . | . | . | . | . | L | . | . | . | . | . | . | . | . | . | . | . | . | . | . | . | . | - | S |
| A/NEW_CALEDONIA/9/2004 | . | . | . | . | . | . | . | . | . | . | L | . | . | . | . | K | . | . | . | . | . | . | . | . | . | . | . | - | S |
| A/NEW_CALEDONIA/20/99 | . | . | . | . | . | . | . | . | . | . | L | . | . | . | . | . | . | . | . | . | . | . | . | . | T | . | . | - | S |
| A/PHILIPPINES/673/2006 | . | . | . | . | . | . | . | . | . | . | L | . | . | . | . | K | T | . | . | H | . | . | K | . | . | . | . | - | S |
| A/SHENZHEN/227/95 | . | . | . | R | T | . | . | . | . | . | . | F | . | . | A | . | . | . | . | . | . | . | . | . | K | . | . | K | . |
| A/SICHUAN/4/88 | . | . | . | R | . | . | . | I | . | . | . | F | Q | K | A | . | . | . | . | . | . | . | . | . | . | . | . | K | . |
| A/SINGAPORE/6/86 | . | . | . | R | . | . | . | I | . | . | . | F | . | K | A | . | . | S | . | . | . | . | . | . | . | . | . | K | . |
| A/SINGAPORE/14/2004 | . | . | . | . | . | . | . | . | . | . | L | . | . | . | . | . | S | . | . | . | T | . | . | . | . | . | . | - | S |
| A/SOLOMON_ISLANDS/03/2006 | . | . | . | . | . | . | . | . | . | . | L | . | R | . | . | K | . | . | . | H | . | . | . | . | . | . | T | - | S |
| A/SOUTH_DAKOTA/06/2007 | N | . | R | . | . | . | . | . | . | . | L | . | . | . | . | K | . | . | . | H | . | L | . | . | . | . | . | - | S |
| A/TAIWAN/1/86 | . | . | . | R | . | . | . | I | . | . | . | F | . | K | A | . | . | S | . | . | . | . | . | . | . | . | . | K | . |
| A/TEXAS/36/91 | . | . | . | R | . | . | . | . | K | . | . | F | . | . | A | . | . | . | . | . | . | . | . | . | . | . | . | K | . |
| A/USSR/90/77 | . | . | . | R | . | K | N | I | . | . | . | F | . | K | A | . | . | S | . | . | . | . | . | R | K | N | . | R | . |
| A/VICTORIA/500/2006 | . | . | . | . | . | . | . | . | . | . | . | . | . | . | . | K | . | . | . | H | . | . | . | . | . | . | . | - | S |
| A/VIRGINIA/01/2006 | . | . | . | . | . | . | . | . | . | . | L | . | . | . | . | K | . | . | . | H | . | . | . | . | . | . | . | - | S |
| A/ENGLAND/333/80 | . | . | . | R | . | K | . | I | . | . | . | F | . | K | A | . | . | S | . | . | . | . | . | . | K | N | . | R | . |
| A/CHILE/4795/00 | . | . | . | R | T | . | . | I | . | . | . | F | . | . | A | . | . | . | K | . | . | . | D | . | . | . | . | K | . |
| A/FUJIAN/156/00 | . | . | . | . | . | . | . | . | . | . | L | . | . | . | . | . | . | . | . | . | . | . | . | . | . | . | . | - | S |
| A/HONG KONG/1870/2008 | . | N | . | . | K | . | . | . | . | G | L | . | . | . | . | R | . | . | . | H | . | . | . | . | . | . | . | - | S |
| A/MALAYSIA/100/2006 | . | . | . | . | . | . | . | . | . | . | L | . | R | . | . | K | . | . | . | H | . | . | . | . | . | . | . | - | S |
| A/MOSCOW/13/98 | . | . | . | R | T | . | . | I | . | . | . | F | . | . | A | . | . | . | . | . | . | . | . | . | . | . | . | K | . |
| A/NEIMENGGU/52/2002 | . | . | . | . | . | . | . | . | . | . | L | . | . | . | . | . | . | . | . | . | . | . | . | . | . | . | . | - | S |

*^a^* Results are reported as amino acid differences between the HA1 sequences of the A/WUHAN/371/95 strain and that of the other virus strains.

*^b^* gap.

*^c^* no change.

**S3 Table (continued).** **Amino acid changes in HA1 of H1N1 seasonal influenza virus from 1977 to 2008.**

| **Virus strain** | **Amino acid position in HA1** | | | | | | | | | | | | | | | | | | | | | | | |
| --- | --- | --- | --- | --- | --- | --- | --- | --- | --- | --- | --- | --- | --- | --- | --- | --- | --- | --- | --- | --- | --- | --- | --- | --- |
|  | 134 | 135 | 139 | 141 | 146 | 153 | 157 | 160 | 163 | 166 | 168 | 170 | 183 | 185 | 186 | 187 | 189 | 190 | 191 | 193 | 194 | 202 | 205 | 207 |
| A/WUHAN/371/95 | A | S | N | K | R | E | L | N | N | V | N | E | P | I | G | N | R | A | I | H | T | V | H | S |
| A/BAYERN/7/95 | . | . | . | . | K | . | . | . | K | . | . | . | S | . | . | D | . | . | . | . | . | . | . | . |
| A/BEIJING/262/95 | . | . | . | . | . | . | . | . | . | . | . | . | S | . | . | D | . | . | . | . | . | . | . | . |
| A/BRAZIL/11/78 | . | . | K | . | . | . | S | . | K | . | . | . | S | . | E | D | K | T | . | R | K | . | N | N |
| A/BRISBANE/59/07 | . | . | . | E | . | G | . | . | K | A | . | . | . | . | . | D | K | . | L | . | . | . | . | . |
| A/BRISBANE/193/2004 | . | . | . | . | . | G | . | . | K | A | . | . | . | . | . | D | . | . | L | . | . | . | . | . |
| A/CAMBODIA/0371/2007 | . | . | . | E | K | G | . | . | K | A | . | . | . | . | . | V | M | T | L | . | K | . | . | . |
| A/CHILE/1/83 | . | A | K | . | . | . | S | . | K | . | . | . | S | . | E | D | K | T | . | R | K | . | . | N |
| A/FLORIDA/13/07 | . | . | . | E | . | G | . | . | K | A | . | . | . | . | . | D | K | . | L | . | . | . | . | . |
| A/FUKUSHIMA/141/2006 | . | . | . | E | K | G | . | . | K | A | . | . | . | . | . | . | . | . | L | . | . | . | . | . |
| A/HONG_KONG/2652/2006 | . | . | . | E | K | G | . | . | K | A | . | . | . | . | . | . | M | T | L | . | K | . | . | . |
| A/INDIA/6263/80 | . | . | K | . | . | . | S | . | K | . | . | . | S | . | E | . | K | T | . | R | K | . | . | N |
| A/JIANGXI/160/2005 | . | . | . | E | . | G | . | . | K | A | . | . | . | . | . | D | . | . | L | . | . | . | . | . |
| A/JOHANNESBURG/82/96 | . | . | . | . | K | . | . | . | K | . | . | . | S | . | . | D | . | . | . | . | . | . | . | . |
| A/KENTUCKY/1/2005 | . | . | . | . | . | G | . | . | K | A | . | . | . | . | R | D | . | . | L | . | . | . | . | . |
| A/KENTUCKY/02/2006 | . | . | . | . | . | G | . | . | K | A | . | . | . | . | . | . | . | . | L | . | . | . | . | . |
| A/NEW_CALEDONIA/9/2004 | . | . | . | . | K | G | . | . | K | A | . | . | . | . | . | G | . | . | L | . | . | . | . | . |
| A/NEW_CALEDONIA/20/99 | . | . | . | . | . | G | . | . | K | . | . | . | . | . | . | D | . | . | L | . | . | . | . | . |
| A/PHILIPPINES/673/2006 | . | . | . | E | K | G | . | . | K | A | . | . | . | . | . | D | K | . | L | . | . | . | . | . |
| A/SHENZHEN/227/95 | . | . | . | . | K | . | . | . | K | . | . | . | S | . | . | D | . | . | . | . | . | . | . | . |
| A/SICHUAN/4/88 | . | . | K | R | . | . | . | . | K | . | . | . | S | . | . | . | . | . | . | . | . | . | . | N |
| A/SINGAPORE/6/86 | . | . | K | R | . | K | S | . | K | . | . | . | S | . | . | D | . | . | . | . | . | . | . | N |
| A/SINGAPORE/14/2004 | . | . | . | . | K | G | . | . | K | A | . | . | . | . | . | D | . | . | L | . | . | . | . | . |
| A/SOLOMON_ISLANDS/03/2006 | . | . | . | E | K | G | . | . | K | A | . | . | . | . | . | D | . | . | L | . | K | . | . | . |
| A/SOUTH_DAKOTA/06/2007 | . | . | . | E | . | G | W | . | K | A | . | . | . | . | . | D | K | . | L | . | . | . | . | . |
| A/TAIWAN/1/86 | . | A | K | . | . | . | S | . | K | . | . | . | S | . | . | D | . | . | . | . | . | . | . | N |
| A/TEXAS/36/91 | T | . | . | E | . | K | . | . | K | . | . | . | S | . | . | D | . | . | . | . | . | . | . | . |
| A/USSR/90/77 | . | . | K | . | . | . | S | . | K | . | . | . | S | . | E | D | K | T | . | R | K | . | N | N |
| A/VICTORIA/500/2006 | . | . | . | . | K | G | . | . | K | A | . | K | . | . | . | D | . | . | L | . | . | . | . | . |
| A/VIRGINIA/01/2006 | . | . | . | . | K | G | . | . | K | A | . | . | . | . | . | D | . | . | L | . | . | . | . | . |
| A/ENGLAND/333/80 | . | . | K | . | . | . | S | S | K | . | . | . | S | . | E | D | K | T | . | R | K | . | . | N |
| A/CHILE/4795/00 | . | . | . | . | K | G | . | . | K | . | K | G | S | M | . | D | . | . | . | . | K | L | . | . |
| A/FUJIAN/156/00 | . | . | . | . | . | G | . | . | K | A | . | . | . | . | . | . | . | . | L | . | . | . | . | . |
| A/HONG KONG/1870/2008 | . | . | . | E | K | G | . | . | K | A | . | . | . | . | . | . | M | T | L | . | K | . | . | . |
| A/MALAYSIA/100/2006 | . | . | . | . | K | G | . | . | K | A | . | . | . | . | . | D | . | . | L | . | . | . | . | . |
| A/MOSCOW/13/98 | . | . | . | . | K | . | . | . | K | . | . | K | S | . | . | D | . | . | . | . | . | . | . | . |
| A/NEIMENGGU/52/2002 | . | . | . | . | . | G | . | . | K | A | . | K | . | . | . | . | . | . | L | . | . | . | . | . |

**S3 Table (continued).** **Amino acid changes in HA1 of H1N1 seasonal influenza virus from 1977 to 2008.**

| **Virus strain** | **Amino acid position in HA1** | | | | | | | | | | | | | | | | | | | | | | | |
| --- | --- | --- | --- | --- | --- | --- | --- | --- | --- | --- | --- | --- | --- | --- | --- | --- | --- | --- | --- | --- | --- | --- | --- | --- |
|  | 209 | 216 | 222 | 224 | 237 | 244 | 252 | 253 | 255 | 258 | 262 | 267 | 269 | 271 | 272 | 273 | 274 | 277 | 283 | 295 | 305 | 310 | 315 | 321 |
| A/WUHAN/371/95 | R | K | D | E | G | A | W | Y | F | S | G | T | N | P | M | N | E | A | Q | V | K | T | V | I |
| A/BAYERN/7/95 | . | . | . | . | . | . | . | . | . | . | . | . | . | S | . | G | . | . | . | . | . | . | . | . |
| A/BEIJING/262/95 | . | . | G | . | . | . | . | . | . | . | . | . | . | . | . | . | . | . | . | . | . | . | . | . |
| A/BRAZIL/11/78 | . | . | G | . | . | . | . | . | . | . | . | . | . | S | . | D | . | T | . | . | . | . | . | . |
| A/BRISBANE/59/07 | K | . | . | . | . | . | R | . | . | . | . | N | . | . | . | D | K | . | . | . | . | A | . | . |
| A/BRISBANE/193/2004 | . | . | N | . | . | . | R | F | . | . | . | . | . | . | . | D | . | . | . | . | . | A | A | . |
| A/CAMBODIA/0371/2007 | K | . | . | . | . | . | R | . | . | . | . | N | . | . | . | D | . | . | . | . | . | A | . | . |
| A/CHILE/1/83 | . | . | N | . | . | . | . | . | . | . | . | . | . | S | . | D | . | . | . | . | . | . | . | . |
| A/FLORIDA/13/07 | K | . | . | . | . | . | R | . | . | . | . | N | . | . | . | D | K | . | . | . | . | A | . | . |
| A/FUKUSHIMA/141/2006 | K | . | . | . | . | . | R | . | . | . | . | N | . | . | . | D | . | . | . | . | . | A | . | . |
| A/HONG_KONG/2652/2006 | K | . | . | . | . | . | R | . | . | . | . | N | . | . | . | D | . | . | . | . | . | A | . | . |
| A/INDIA/6263/80 | . | . | . | . | . | . | . | . | . | . | . | . | . | S | I | D | . | . | . | . | . | . | . | . |
| A/JIANGXI/160/2005 | . | . | . | . | . | . | R | F | . | . | . | . | . | . | . | D | . | . | . | . | . | A | A | T |
| A/JOHANNESBURG/82/96 | . | . | G | . | . | . | . | . | . | . | . | . | . | S | . | G | . | . | . | . | N | . | . | . |
| A/KENTUCKY/1/2005 | . | . | . | . | . | . | R | F | . | . | . | . | . | . | . | D | . | . | . | . | . | A | A | . |
| A/KENTUCKY/02/2006 | . | . | . | . | . | . | R | F | . | . | . | . | . | . | . | D | . | . | . | . | . | A | . | . |
| A/NEW_CALEDONIA/9/2004 | K | . | . | . | . | . | R | . | . | . | . | N | . | . | . | D | . | . | . | . | . | A | . | . |
| A/NEW_CALEDONIA/20/99 | . | . | . | . | . | . | . | . | . | . | . | . | . | . | . | D | . | . | . | . | . | A | . | . |
| A/PHILIPPINES/673/2006 | K | . | . | . | . | T | R | . | . | . | . | N | . | . | . | D | . | . | . | . | . | A | . | . |
| A/SHENZHEN/227/95 | . | . | G | . | . | . | . | . | . | . | . | . | T | S | . | G | . | . | . | . | . | . | . | . |
| A/SICHUAN/4/88 | . | . | . | . | . | . | . | . | . | . | . | . | . | S | . | D | . | V | . | . | . | A | . | . |
| A/SINGAPORE/6/86 | . | . | . | . | . | . | . | . | . | . | . | . | . | S | . | D | . | . | . | . | . | . | . | . |
| A/SINGAPORE/14/2004 | . | . | . | . | . | . | R | . | . | . | . | . | . | . | . | D | . | . | H | . | . | A | . | . |
| A/SOLOMON_ISLANDS/03/2006 | K | . | . | . | . | . | R | . | . | . | . | N | . | . | . | D | . | . | . | . | . | A | . | . |
| A/SOUTH_DAKOTA/06/2007 | K | . | . | . | . | . | R | . | . | . | . | N | . | . | . | D | K | . | . | . | . | A | . | . |
| A/TAIWAN/1/86 | . | . | G | . | E | . | . | . | . | . | . | . | . | S | . | D | . | . | . | . | . | . | . | . |
| A/TEXAS/36/91 | . | . | G | . | . | . | . | . | . | . | . | . | . | S | . | D | . | . | . | . | . | . | . | . |
| A/USSR/90/77 | . | E | G | A | . | . | . | H | . | N | . | . | . | S | . | D | . | T | . | I | . | . | . | . |
| A/VICTORIA/500/2006 | K | . | . | . | . | . | R | . | L | . | . | N | . | . | . | D | . | . | . | . | . | A | . | . |
| A/VIRGINIA/01/2006 | K | . | . | . | . | . | R | . | . | . | . | N | . | . | . | D | . | . | . | . | . | A | . | . |
| A/ENGLAND/333/80 | . | . | G | . | . | . | . | . | . | . | . | . | . | S | . | D | . | . | . | . | . | . | . | . |
| A/CHILE/4795/00 | . | . | . | . | . | . | . | . | . | . | . | I | . | S | . | G | . | . | . | . | . | . | . | V |
| A/FUJIAN/156/00 | . | . | . | . | . | . | R | . | . | . | . | . | . | . | . | D | . | . | . | . | . | A | . | . |
| A/HONG KONG/1870/2008 | K | . | . | . | . | . | R | . | . | . | . | N | . | . | . | D | . | . | . | . | . | A | . | . |
| A/MALAYSIA/100/2006 | K | . | . | . | . | . | R | . | . | . | . | N | . | . | . | D | . | . | H | . | . | A | . | . |
| A/MOSCOW/13/98 | . | . | N | . | . | . | . | . | . | . | E | . | . | S | . | G | . | . | . | . | . | . | . | . |
| A/NEIMENGGU/52/2002 | . | . | . | . | . | . | R | . | . | . | . | . | . | . | . | D | . | . | . | . | . | A | . | . |
